# Needle-free intradermal vaccination, an opportunity to improve commercial pig welfare

**DOI:** 10.1017/awf.2024.53

**Published:** 2024-11-04

**Authors:** Isabel Lewis, Harriet Wishart, Ellie Breeze, Poppy Setter, Jonathan Amory

**Affiliations:** 1 Anglia Ruskin University, Chelmsford Campus, Lordship Road, Chelmsford, Essex CM1 3RR, UK; 2 Hartpury University, Hartpury, Gloucester GL19 3BE, UK

**Keywords:** animal welfare, behaviour, needle-free vaccination, pain, piglets, vocalisations

## Abstract

In-farm livestock production vaccinations are commonly delivered intramuscularly using needles. While there are alternative strategies these have been subject to little attention and limited commercialisation. One such alternative is needle-free vaccines and studies have focused on the immune response few have addressed the welfare implications. This study aims to compare the impact of intradermal needle-free vaccination and intramuscular injection in terms of the welfare of the piglets. A total of 179 piglets were divided into two treatments: intradermal needle-free delivery and intramuscular delivery of a vaccine. Measures of health and welfare included, vocalisations, behavioural observations, papule formation, and weight. Piglets vaccinated via the needle-free intradermal route vocalised less and displayed no significant behavioural differences but showed increased weight compared to piglets vaccinated intramuscularly. The use of a needle-free device to deliver a vaccine through an intradermal route revealed no adverse effects on piglet welfare and supports the use of alternative strategies to vaccinate livestock.

## Introduction

The global human population is predicted to rise to 11.2 billion by 2100, according to the United Nations, leading to increasing demand for animal-derived food production. Over recent years the welfare of farmed livestock species has been placed at the forefront for consumers and farmers (Fernandes *et al.* 2021; Vigors *et al.* 2021). The pursuit of optimum animal welfare and the desire for livestock animals to ‘experience positive lives’ (Vigors *et al.* 2021) has driven researchers and producers to seek to improve the welfare of farmed animals (Alonso *et al.* 2020; Lucas *et al.* 2023). In the UK there are over 10,000 pig holdings as of 2021, with 60% of UK sows farmed indoors (Woods 2019; AHDB 2024).

Vaccination is commonly used as a preventative strategy against disease and pigs are typically vaccinated during their production lifecycle (Gebhardt *et al.* 2020; Temple *et al.* 2020). Delivery of vaccines is commonly achieved via intramuscular (IM) injection (Có-Rives *et al.* 2023), however, concerns exist over the use and reuse of needles to deliver these vaccines. Best-practice would see needles changed between use on each individual animal however the reuse of needles is commonplace within the industry (AHDB 2019). A recent study by Owen *et al.* (2022) revealed that 81% of farmers surveyed reuse the needle and there was variation in the frequency of needle change. In addition, the study demonstrated that needles become damaged after 12 uses. Therefore, a welfare concern of the IM method is the pain caused by the procedure, especially when needles are reused. Another concern is the potential of infection being spread between pigs. For example, porcine reproductive and respiratory syndrome virus (PPRSV) has been shown to spread to susceptible pigs via contaminated needles (Pileri & Mateu 2016). Thirdly, a public health issue is that broken needles can be left in the pig carcases leading to food health and safety issues (Imeah *et al.* 2020). Whilst alternatives for IM vaccinations exist, such as intranasal or oral vaccines, there are limited options that are commercially available (Temple *et al.* 2020).

Another alternate method is intradermal (ID) vaccination, which can be achieved using needle-free devices (NFDs) and has been reported to reduce the negative effects imposed by needle syringes (Có-Rives *et al.* 2023). Multiple vaccines are now available to be delivered using NFDs, and studies have confirmed humoral and cell-mediated immune responses (Cho *et al.* 2022; Madapong *et al.* 2021; Yang *et al.* 2020). Porcine circovirus type 2 (PCV2) and *Mycoplasma hyopneumoniae* are two challenging porcine pathogens for which vaccinations are highly effective (Yang *et al.* 2020) and MHYOSPHERE® PCV ID (Hipra laboratories, Spain) is the first vaccine that can be administered intradermally to protect against both pathogens (Puig *et al.* 2022).

A systematic review conducted by Có-Rives *et al.* (2023) identified that 35% of skin-based inoculations use NFDs (jet injectors). Whereas 17.9 and 10.7% used needles/syringes or microneedles, respectively, and 18.6% of studies did not specify the device used which may increase the percentage using a needle-based approach (Có-Rives *et al.* 2023). This indicates a recent upsurge in the usage of ID NFDs, with commercial devices namely the IntraDermal Application of Liquids (IDAL) (Merck, Germany) and the Hipradermic (HIPRA, Spain) available.

There are multiple advantages of NFDs as removing needles from the equation can avoid issues previously highlighted. In one study, Salman *et al.* (2023) demonstrated minimising disease transmission specifically, African swine fever virus was unable to be transmitted by using the IDAL instead of IM needles. Positive welfare benefits for pigs have also been discovered, for instance, studies have reported NFD causing a reduction in pain and aversive behaviour (Temple *et al.* 2017, 2020; Scollo *et al.* 2020; Dalmau et al. 2021).

With a growing interest in NFD and new commercially available devices entering the market, it is essential that claims about improving welfare are substantiated. This study, therefore, aims to explore the welfare (as measured through pig behaviour) benefits of a previously unreported ID NFD on a commercial UK pig unit.

## Materials and methods

### Ethical statement

The experimental protocols described in the study were approved (2022-1621) by the ethical review panel at ARU Writtle, UK.

### Animal, housing and experimental procedure

A total of 179 piglets (crossbreed with genetic make-up of 50% Large White, 25% Landrace, and 25% Pietrain) on a commercial farm in Essex, UK were included in this study and either vaccinated at four weeks of age via an ID NFD or by an IM method. The study was repeated over two trials: Trial 1 included 91 piglets (46 vaccinated via ID NFD procedure and 45 by IM) in six pens (three for each treatment); and Trial 2 included 88 piglets (44 ID NFD and 44 IM) which used four pens (two for each treatment). Litters were weaned at approximately 28 days of age and separated into pens (2.5 × 3.6 m; length × width), in groups of 18–20 piglets balanced for weight. Each pen had plastic slatted floors, water was provided from nipple feeders and pigs were fed *ad libitum.* Enrichment items such as a rope and plastic toys were provided in each pen.

The pigs were split into two treatments: (i) Pigs vaccinated against Porcine Circovirus type 2 (PCV2) with a 1 mL intramuscular vaccination with Ingelvac CircoFLEX® (Boehringer Ingelheim; Germany) (IM) a water-based vaccine expressing ORF2 antigen of PCV2; and (ii) Pigs vaccinated against PCV2 and *Mycoplasma hyopneumoniae* with 0.2 mL Mhyosphere® (Hipra Laboratories, Spain). An oil-based vaccine that contains inactivated recombinant *Mycoplasma hyopneumoniae* and the capsid protein of PCV2 delivered via an ID NFD, specifically the Hipradermic® 3.0, a battery-powered device.

There were a total of ten pens, with five pens per treatment. Vaccinations were completed in the morning by trained persons and all pigs were handled in the same manner to administer the vaccines. In brief, one person’s role was to hold the piglet for the ID group whilst another person used the NFD, and IM piglets were handled and vaccinated by the same person. The vaccines for both treatments were administered in the neck region. This study did not include a non-vaccinated control group since our objective was to observe the behaviour exhibited by piglets vaccinated using two different routes.

### Behavioural measures

The general activity of the piglets was measured using instantaneous scan sampling at 1 and 24 h post-vaccination (after the last pig was vaccinated), using an ethogram adapted from the Dalmau *et al* (2021) study (Table 1). Behaviours were recorded every 6th min during a period of 180 min for each pen and a total of 600 observations were taken. Each pen was recorded using an H.265 4MP Eyeball PoE infrared dome camera (Genie, WIP4BV5) installed by Clearview (UK). The videos were recorded onto a 3 TB hard drive-equipped H.265 eight-channel network video recorder (Genie, WNVR185) (Clearview 2023). All recorded videos per pen were watched and behaviours recorded by two trained observers. To avoid bias, the videos were split between the two observers and balanced across the two treatments. In addition, the incidence of piglet vocalisation during vaccination was recorded on an individual level at point of vaccination prior to pen allocation on a yes or no basis. Observers were not blinded in this study.

**Table 1.** **The ethogram used for scan sampling piglet behaviour adapted from Dalmau *et al.* (** 2021 **)**

Behaviour	Definition
Standing	Piglet standing upright on all four feet without moving
Laying sternally	Piglet not bearing weight on either the right or left side of the body when laying down (sternum touching the ground vertically)
Laying right side	Piglet is bearing weight on the right side when laying down
Laying left side	Piglet is bearing weight on the left side when laying down
Locomotion	Piglet standing on all four feet while moving in a forward direction around the pen
Manipulation fixtures	Piglet is biting, chewing, or destroying the pen (etc, chewing the edges of the pen)
Sitting	Piglet is resting on its hindquarters in a sitting position
Escape attempt	Piglet is standing on the back legs while raising front legs on the side or front of the pen
Tail and ear directing	Piglet is chewing or biting another piglet’s tail or ear
Playing	Piglet is interacting, moving or chewing an enrichment toy provided
Drinking and Feeding	Piglet is seen eating feed from the feeder or using getting water by the nipple drinkers

### Physical measurements

To assess the impact of the vaccines on growth, piglets were weighed on the day of vaccination and one week post-vaccination (at approximately five weeks of age). The incidence of papules was also assessed one week post-vaccination (papule formation denotes successful delivery of vaccine into the dermis layer; Bik *et al.* 2022).

### Statistical analysis

Statistical analysis was completed using GenStat 22^nd^ edition (VSNi). Trial did not have an effect on observations, therefore the five pens of piglets per treatment was considered the replicate within the analysis. The effect of vaccine type and time after vaccination (including interactions between these) on behaviours was analysed via a two-way ANOVA. Counts of observations for each behaviour were summed for each observation period, then the incidence of behaviour as a percent of the total possible observations was used in analysis. The following behaviours were combined prior to statistical analysis due to low instances of observation: ‘Side-lying’ combined observed behaviours of ‘lying right’ and ‘lying left’; ‘Moving’ combined ‘locomotion’, ‘manipulating fixtures’, ‘playing’ and ‘escape attempt’; and ‘Standing or sitting’ combined ‘standing’ and ‘sitting’. Chi-squared tests comparing vaccine type were carried out on the counts of whether piglets vocalised at the point of vaccination. We used *t*-tests to compare the averages of piglet weights at vaccination and one week post-vaccination between the two vaccination types.

## Results

### Behavioural observations

The piglets showed no significant difference in behaviours observed between the two vaccine delivery methods (Table 2). Piglets were observed spending more time drinking and feeding at 24 h compared to 1 h post-vaccination (*P* < 0.001).

**Table 2.** The percentage of piglet behaviours recorded at two scan sampling timepoints (1 h and 24 h) post-vaccination with either an Intradermal (ID) or Intramuscular (IM) vaccine. Standard error of difference (SED) was calculated between the vaccine type and time to show effect size.

Behaviour (% observed)	ID (n = 90)		IM (n = 89)				
	1 hr	24 hr	1 hr	24 hr	Vaccine SED^1^	Time SED^1^	V × T SED^1^
Laying sternally	41.70	31.50	39.60	33.50	4.72	4.72	6.68
Side Lying	8.06	7.50	11.17	8.29	1.36	1.36	1.93
Moving	24.60	24.80	26.50	20.00	2.66	2.66	3.76
Tail and Ear Biting	1.47	2.16	1.81	4.26	0.75	0.75	1.07
Drinking or Feeding	2.79	10.73	2.26	12.47	1.42	1.42***	2.01
Standing or Sitting	21.40	23.30	18.60	21.40	4.65	4.65	6.58

1 SED = Standard Error of Difference.

*** indicates significance at *P* < 0.001.

**Figure 1.**
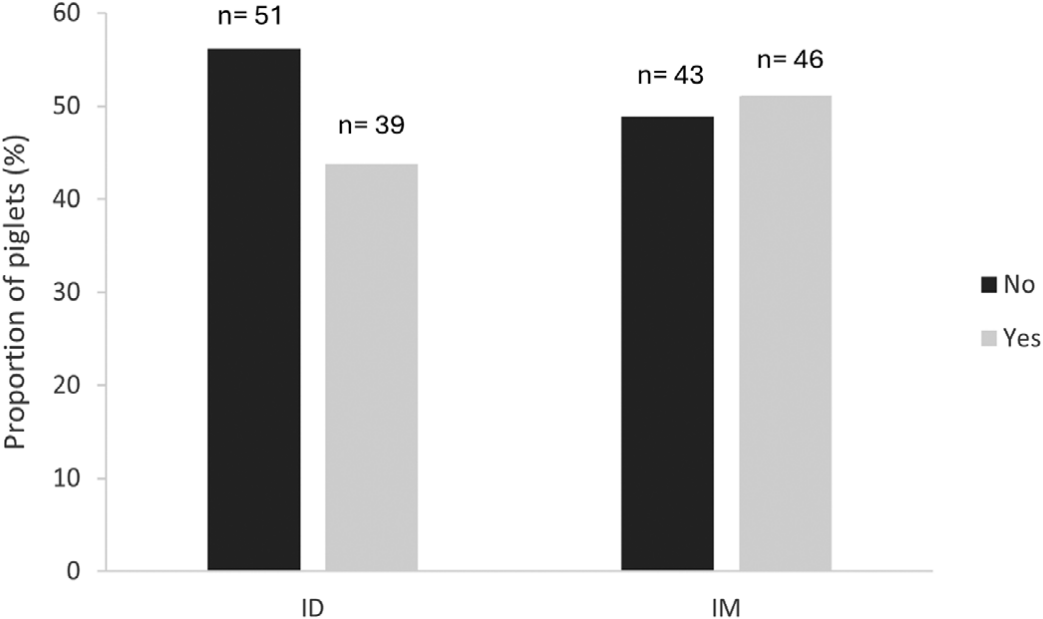
The proportion (%) of piglets (n = 179) that vocalised (‘Yes’ [light grey]) compared to those that did not (‘No’ [dark grey]) at the time of vaccination in each treatment group. ID: Intradermal; IM: Intramuscular.

There was no significant difference in the proportion of piglets that vocalised during IM compared to ID vaccination (51% and 43%, respectively; *P* = 0.329; Figure 1).

### Physical measurements

**Figure 2.**
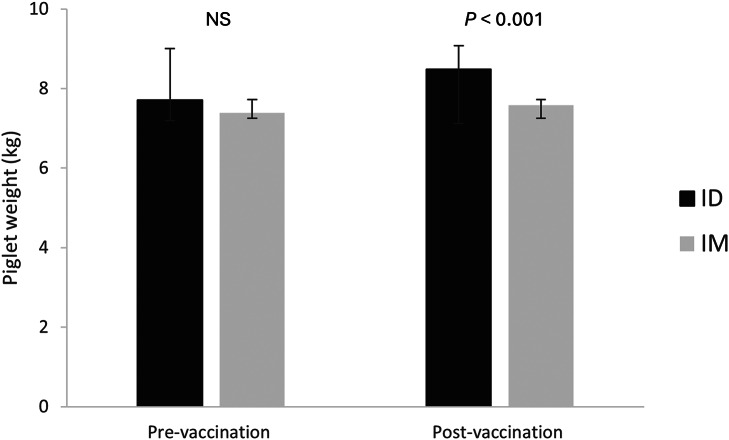
Mean (± SD) piglet (n = 179) weights (kg) at pre-vaccination (left) at four weeks of age and (right) post-vaccination at five weeks of age for each treatment group (ID: intradermal [dark grey], IM: intramuscular [light grey]). A *t*-test was performed and showed non-significance (NS) and significance indicated at *P* < 0.001.

At the start of the trials all piglets were weighed pre-vaccination and were at a similar average weight between ID: 7.71 (± 1.64) kg and IM: 7.39 (± 1.79) kg. However, ID piglets were significantly heavier one week post-vaccination compared to IM piglets (8.49 [± 1.79] kg and 7.58 [± 1.79] kg, respectively; *P* < 0.001; Figure 2). Papules were noted after the vaccine (< 1 h) was given in the ID piglets however there was no evidence of papules after seven days.

## Discussion

This study revealed minimal differences in piglet behaviour and welfare between the two different routes of vaccination. It follows on from similar studies on piglets that were in different settings (either experimental or commercial) (Temple *et al.* 2017, 2020; Dalmau *et al.* 2021; Puig *et al.* 2022; Salman *et al.* 2023) with this study using pigs in a UK commercial setting.

The piglets’ behavioural response was assessed through scan sampling and vocalisations as per previous work (Dalmau *et al.* 2021). Vocalisation at the time of vaccination has been measured as an indicator of pain. A similar percentage of pigs (55%) did not vocalise at the point of vaccination in Scollo *et al.*’s (2020) study using the IDAL to deliver Porcilis® *(M.hyopneumoniae*; MSD Animal Health, US) and Porcilis® (PCV2; MSD Animal Health). While Temple *et al.*’s (2020) study only focused on high-pitched vocalisations as a sign of stress and noted 25% more piglets vocalised in the IM group compared to the control or ID. Analysing the pitch and length of vocalisation could provide additional evidence to support the welfare benefits of ID vaccinations and should be considered in the future.

Regarding the scan sampling of piglets, no behaviour showed a significant treatment effect across the two time-points which was in accordance with the findings of Dalmau *et.al* (2021). However, a minimal effect of treatment on different lying positions was reported which differs from previous findings. Dalmau *et al.* (2021) used UNISTRAIN® (Hipra, Spain) to vaccinate for porcine reproductive and respiratory syndrome (PRRS) as well as environmental differences which may explain the differences in responses observed. Previous research has indicated that vaccines have different reactogenicity profiles based on the inflammatory response which can be influenced by the adjuvant type, antigen dose and physicochemical properties (Hervé *et al.* 2019). The significant increase in piglets eating and drinking at 24 h compared to 1 h post-weaning was seen within both treatments and likely not an effect of the vaccine but instead a result of acclimatisation to the new environment. No adverse behavioural effects were demonstrated in piglets vaccinated using the ID route which is a positive outcome suggesting no drop in welfare compared to the industry-wide, currently accepted IM method. This can be used to promote the uptake of NFDs and encourage future research into other possible benefits.

Papule formation, as noted earlier, is a local response from ID vaccination and indicates successful delivery of the vaccine into the dermis layer (Bik *et al.* 2022). The literature has reported visible papules from 21 to 28 days post-vaccination. However, here, papules resolved by day 7 post-vaccination and are in agreement with previous reports that this is not an adverse effect and does not influence any behaviour. Piglets in the ID treatment group one week post-vaccination were heavier compared to the IM group (*P* < 0.001). This is in accordance with previous findings where the avoidance of invasive and stressful procedures, such as invasive surgical procedures, has been associated with weight gain (Morgan *et al.* 2019). Also, Puig *et al.* (2022) reported that replacing IM vaccines with the Mhyosphere® delivered ID resulted in a higher growth performance in the first weeks post-weaning. In this study, weight gain could be considered both a welfare and a production benefit.

### Animal welfare implications

This study revealed the ID NFD to not cause any negative impacts on the piglets’ welfare and, while not empirically evidenced, it could be presumed the lack of injection improved the piglets’ vaccination experience, thereby improving their welfare. This supports the literature claiming that ID NFDs offer an alternative that benefits animal welfare (Temple *et al.* 2017, 2020; Scollo *et al.* 2020; Dalmau *et al.* 2021; Có-Rives *et al.* 2023). Future studies should aim to evaluate the potential benefits of the device, such as the reduction in labour and promotion of health to both piglets and farmers. To promote high quality animal welfare, ID NFDs such as the Hipradermic 3.0 should be further investigated in commercial farm settings and promoted for future use.
